# Elevated matrix metalloproteinase 7 expression promotes the proliferation, motility and metastasis of tongue squamous cell carcinoma

**DOI:** 10.1186/s12885-020-6521-4

**Published:** 2020-01-14

**Authors:** Shuo Yuan, Li-song Lin, Rui-Huan Gan, Li Huang, Xiao-ting Wu, Yong Zhao, Bo-hua Su, Dali Zheng, You-Guang Lu

**Affiliations:** 1 Department of Preventive Dentistry, School and Hospital of Stomatology, Fujian Medical University, 246 Yang Qiao Middle Road, Fuzhou, 350000 China; 2 Key Laboratory of Ministry of Education for Gastrointestinal Cancer, Fujian Medical University, 1 Xue Yuan Road, University Town, Fuzhou, 350122 China; 3 Department of Oral and Maxillofacial Surgery, Affiliated First Hospital of Fujian Medical University, 20 Cha Zhong Road, Fuzhou, 350005 China; 4 Key laboratory of Stomatology of Fujian Province, School and Hospital of Stomatology, Fujian Medical University, 88 Jiaotong Rd, Fuzhou, 350004 China

**Keywords:** Tongue cancer, MMP7, Proliferation, Metastasis

## Abstract

**Background:**

Matrix metalloproteinase 7 (MMP7), as the smallest member of the matrix metalloproteinase family, has been verified to be implicated in cancer progression, especially metastasis. However, its expression pattern and function in tongue cancer is not clear.

**Methods:**

The expression of MMP7 in human tongue squamous cell carcinoma (TSCC) specimens compared with their respective paired nontumour tissues by real-time PCR and immunohistochemical staining. The effect of MMP7 on the proliferation, apoptosis, migration, invasion of tongue cancer cells was tested in appropriate ways after MMP7 siRNA knockdown or overexpression. The effect of MMP7 on lymph node metastasis in vivo was analyzed using a high-metastasis orthotopic nude mouse tongue transplanted tumour model.

**Results:**

We found markedly elevated expression of MMP7 in human TSCC specimens compared with their respective paired nontumour tissues, and this high expression was correlated with the patients’ lymph node metastasis. Furthermore, the results of molecular functional assays confirmed that MMP7 promotes cell proliferation, migration and invasion of TSCC cells. Knockdown of MMP7 inhibited lymph nodes metastasis in vivo.

**Conclusions:**

MMP7 plays an oncogenic role in carcinogenesis and metastasis of tongue cancer, and may serve as a potential therapeutic target for tongue cancer.

## Background

Approximately 300,000 oral cavity cancer cases were diagnosed in 2012 worldwide, and 145,000 deaths occurred, especially in developing regions (77%) [1]. Tongue squamous cell carcinoma (TSCC) is the most common oral malignant neoplasm and the leading cause of oral cancer-related death. Although some therapeutic advances combined with refined surgical resection operation such as novel chemotherapy and accurate radiotherapy have been achieved, the overall survival of patients with TSCC has remained at the same level due to its invasion, metastasis, drug resistance and recurrence. Thus, it is necessary to identify critical molecular signaling pathways related to TSCC progression that could offer accurate pathogenesis, early diagnosis and targeted treatments to improve clinical outcomes.

Matrix metalloproteinases (MMPs), a large family of zinc- and calcium-dependent endopeptidases comprising 24 members to date, demonstrate the common ability to degrade almost all components of the extracellular matrix (ECM) and basement membranes. Many physiological and pathological roles have been described for MMPs, such as in embryogenesis, inflammation, tissue remodeling (growth, development, and repair), atherosclerosis, and Alzheimer’s disease, when their activities are at modest levels. However, many researchers have focused on the key role of MMPs in carcinogenesis, especially cancer cell invasion and metastasis, because the ECM and basement membranes act as physical barriers to the process of hematogenous and lymph node metastasis, which have been recognized as essential steps in the complicated process of malignant cell movement to the adjacent soft tissue, surrounding muscles and bones or even distant organs. MMPs are abundantly detected in numerous malignant neoplasms and have critical implications in almost all stages of tumour progression [2]. Recent studies have shown that aberrant MMP expression is associated with the invasion and metastasis of several malignant tumours (colorectal [3], prostate [4], liver [5], breast [6], retinoblastoma [7], and lung [8]) both in vitro and *vivo*. Among the MMPs, MMP7 (aka matrilysin1) is the smallest secreted proteolytic enzyme, lacking the C-terminal hemopexin domain compared with other family members, with a wide spectrum of substrate specificity against ECM components, including laminin, type IV collagen, fibronectin, and proteoglycans [9, 10], as well as other molecules, such as E-cadherin, β4 integrin, tumour necrosis factor-α, and the Fas ligand [11]. Interestingly, previous investigations have demonstrated that overexpression of MMP7 may contribute to many malignant tumours, including colorectal cancer [12, 13], pancreatic cancer [14, 15], lung cancer [16], and prostate cancer [17], indicating that MMP7 could be a critical molecular biomarker for oncogenicity.

However, the exact role of MMP7 in TSCC initiation, progression, invasion and metastasis remains unclear, and the mechanisms underlying MMP7 regulation of malignant transformation remain to be elucidated. The aim of our present study was to investigate the expression level of MMP7 and its functional impact on human tongue squamous cell carcinoma. We found that MMP7 was remarkably overexpressed in TSCC compared with that in adjacent nontumour tissues both at the mRNA and protein levels. Functional assays in vitro and in an orthotopic nude mouse transplanted tumour model in vivo revealed that MMP7 can promote tongue cancer cell proliferation, migration, and invasion.

## Methods

### Clinical samples and cell culture

Pathologically diagnosed tissue samples were obtained from the First Affiliated Hospital of Fujian Medical University and Fuzhou General Hospital of Nanjing Military Command. Ninety-two paraffin-embedded tongue cancer samples, without metastasis or recurrence, and their respective adjacent normal tissues, had been kept on-file by the pathology department. Another 53 paired fresh samples were collected to quantify MMP7 mRNA expression. These studies were approved by the Institutional Review Board of Fujian Medical University, and written informed consent was obtained from each participant. The CAL27 and SCC9 cell lines were purchased from ATCC (American Type Culture Collection). The cells were maintained in the suggested medium and were incubated at 37 °C in a humidified atmosphere of 95% air and 5% CO_2_. All cell lines were STR-authenticated annually by Shanghai Biowing Applied Biotechnology Co. LTD, Shanghai, China.

### Quantitative real-time PCR analysis

Total RNA was extracted from the tissues and cells using TRIzol reagent (#15596018; Invitrogen, Carlsbad, CA, USA), and RNA purities and concentrations were detected by ultraviolet spectrometry. The RNAs were separately diluted to the same concentration after being measured and then were reverse transcribed into cDNA using the PrimeScript RT reagent kit (#RR037A; Takara, Japan). PCR was performed in triplicate using the primers listed in Table 1 and SYBR Premix Ex Taq™ (#RR420A; Takara) according to the manufacturer’s instructions. The fluorescence values from the 12 cycles were used as the background signal, and the threshold value was set at 10 times the standard deviation of the fluorescence signals of cycles 4–12. The expression levels were normalized to the β-actin mRNA levels for each sample obtained from parallel assays. The data were analyzed according to the 2^- ΔΔCt^ method.

**Table 1:** Sequences of the primers used in this study

Gene	Accession No.	Forward	Reverse
ACTB	NM_001101	CCTGGCACCCAGCACAAT	GGGCCGGACTCGTCATACT
MMP7	NM_002423	CATGAGTGAGCTACAGTGGGA	CTATGACGCGGGAGTTTAACAT

### Immunohistochemical staining assay

The immunohistochemical SP three-step approach was used to stain and analyze tongue pathological tissues using ovarian carcinomas as a positive control group, and phosphate-buffered saline (PBS) was used instead of the primary antibodies in the negative control groups. After deparaffinization in xylene, the sections were rehydrated in a decreasing gradient of ethanol and washed for 10 min in PBS (pH 7.2). Endogenous peroxidase activity was inhibited by incubation in methanol containing 3% H_2_O_2_ for 10 min. After several washes in PBS, the sections were blocked with a universal blocking reagent (Maxin, USA) for 10 min at room temperature and then were incubated with the primary antibody against MMP7 (1:1500 dilution; ab205525; Abcam, UK) for 1 h at room temperature. After several washes in PBS, the sections were incubated with a biotin-conjugated secondary antibody (Maxin) for 10 min at room temperature. After several washes in PBS, the sections were incubated with streptavidin-peroxidase (Maxin) for 10 min at room temperature. The sections were rinsed with PBS, and the antibody complexes were visualized by incubation with diaminobenzidine tetrahydrochloride (DAB) chromogen (Maxin). The sections were then counterstained with hematoxylin (Dako, Denmark), dehydrated and examined by light microscopy. All slides were reviewed independently by two pathologists who were blinded to each other’s readings. The degree of MMP7 staining was quantified as follows: the score of stained tumour cells (0, no positive cells; 1, ≤30% positive cells; 2, 30–50% positive cells; 3, ≥ 50% positive cells) multiplied by the score of staining intensity (0, no staining; 1, weak staining, light yellow; 2, moderate staining, yellow brown; 3, strong staining, brown) to obtain a final score ranging from 0 to 9. A final score of 0 was classified as the no expression group (−),1–3 as the low expression group (+), and > 4 as the high expression group (++).

### RNAi and plasmid transfection

Sixteen hours before transfection, CAL27 and SCC9 cells in the exponential phase of growth were digested, counted and plated into 6-well plates at 3 × 10^5^ (CAL27) or 1 × 10^5^ (SCC9) cells/well. The cells were then transfected with siRNAs (GenePharma, Shanghai, China; the sequences are indicated in Table 2) using Lipofectamine RNAiMAX (1044526) or plasmid (pCDH-CMV-MCS-EF1-Puro-MMP7, TongYong, Anhui, China) using Lipofectamine 3000 (1713234) (both from Invitrogen) according to the manufacturer’s instructions.

**Table 2:** Sequences of the siRNAs used in this study

Name	Sense	Antisense
NC	5′-UUCUCCGAACGUGUCACGUTT-3′	5′-ACGUGACACGUUCGGAGAATT-3′
siRNA- 208	5′-CCAACAGUUUAGAAGCCAATT − 3′	5′-UUGGCUUCUAAACUGUUGGTT-3’
siRNA- 658	5′-GCAGUCUAGGGAUUAACUUTT − 3’	5′-AAGUUAAUCCCUAGACUGCTT − 3’
siRNA- 720	5′-GGACAUUCCUCUGAUCCUATT −3’	5′-UAGGAUCAGAGGAAUGUCCTT − 3’

### Western blotting

Total cell protein was extracted, and protein assays were performed using a BCA kit and an ELISA reader. Total protein was separated by 10% SDS-PAGE and transferred to PVDF membranes (Amersham, USA). Subsequently, the membranes were immunoblotted with primary antibodies against MMP7 (1:1000 dilution; ab205525; Abcam, UK) or tubulin (1:1000 dilution; CW0098A; KangWei, China) in 5% bovine serum albumin overnight, washed three times with Tris-buffered saline with 0.1% Tween-20, and incubated with secondary antibody (Mouse BA1050; Rabbit BA1054; 1:1000 dilution; Boster, China). The immunoreactive protein bands were visualized using CDP-Star reagent (Roche, USA), and the signals were scanned with a densitometer for semiquantification of the signal intensities.

### Cell viability assay

Cell proliferation was measured by counting the cells in the logarithmic phase using Cell Counting Kit-8 (#CK04; Dojindo Kumamoto, Japan). The cells were first transfected with siRNA or plasmid and then were plated into a 96-well plate. Cells from each group were plated in 3 wells, and each well contained 3 × 10^3^ cells (CAL27) or 2 × 10^3^ cells (SCC9). The absorbance of each well was measured with a microplate reader at the same time over 6 consecutive days. This process was repeated in triplicate for the statistical analyses and to draw the corresponding curves.

### Colony formation assay

Thirty-six hours after siRNA or plasmid transfection, the cells were plated into 6-well plates (800 cells/well) and cultured for 2 weeks. Colonies were fixed with cold methanol for 10 min and stained with 1% crystal violet for 30 min.

### In vitro cell migration assay

The cell migration assays were performed in 24-well Transwell chambers without Matrigel (#353097; Falcon Corning, USA) and with Matrigel (#354480; Falcon Corning, USA). Thirty-six hours after siRNA or plasmid transfection, the cells were plated into the upper chamber at a density of 5.0 × 10^4^ cells/well (CAL27) or 2.0 × 10^4^ cells/well (SCC9) in 500 μl FBS-free DMEM, and then 800 μl of DMEM containing 10% FBS was added to the lower chamber. Forty-eight hours later, the cells in the upper chamber were removed with cotton swabs and stained with 1% crystal violet for 10 min. The cells of five random microscopic fields (× 100) were counted and photographed.

### Wound healing assay

The cells transfected with siRNAs or the pCDH-MMP7 plasmid were seeded into a 6-well plate. The cells grew to monolayers covering the bottom of the plate, 20 μl (CAL27) or 200 μl (SCC9) tips were used to cross-scratch the cell monolayers, then the media were replaced with fetal bovine serum-free DMEM (Dulbecco’s Modified Eagle’s Medium), and finally imaged at a fixed point 0 h and 36 h or 48 h after scratching.

### Establishment of stabilized cell lines

Lentiviral vector constructs of MMP7 (shRNA-208, shRNA-720) corresponding to the negative control (shRNA-NC) were purchased from Shanghai Genechem Co. Ltd., China. Our research group previously isolated a high-metastasis tongue cancer cell line from CAL27 named LN4. LN4 cells were infected by these lentiviral vectors follow the manufacturer’s instructions to establishing the stabilized cell lines, which knocked down MMP7. The effect of knocking down was confirmed by quantitative real-time PCR and Western blotting analysis. The designed and chemically synthesized shRNA sequences were as follows: shRNA-208: Sense 5′-CCGGCCAACAGTTTAGAAGCCAACTCGAGTTGGCTTCT AAACTGTTGGTTTTTG-3′/Antisense 5’AATTCAAAAACCAACAGTTTAGAAGCC AACTCGAGTTGGCTTCTAAACTGTTGG-3′; shRNA-720: Sense 5′-CCGGGGAC ATTCCTCTGATCCTACTCGAGTAGGATCAGAGGAATGTCCTTTTTG-3′/ Antisense5’-AATTCAAAAAGGACATTCCTCTGATCCTACTCGAGTAGGATCAGAG GAATGTCC − 3′.

### Orthotopic xenograft cancer model

The experimental animal protocol was approved by the Animal Care and Use Committee of Hospital of Stomatology, Fujian Medical University. SPF-grade male BALB/c nude mice aged 5–6 weeks were purchased from the Center for Animal Experiments of Fujian Medical University. Nude mice were randomly assigned to three groups, each of which comprised 15 mice. LN4 cells with stable knockdown of MMP7 and control cells (1 × 10^6^) were suspended in 20 μl serum-free DMEM and then were injected into the right axillary fossa of each mouse. The mice were sacrificed and dissected 50 days after injection, and the lymph nodes from the neck, oxter, abdomen and tongue, heart, liver, lung were collected for HE staining (hematoxylin and eosin stain) to determine the presence of cancer cells, which reflects the metastasis ability of the cells in vivo.

### Statistical analyses

The data were analyzed using the SPSS 18.0 statistics software package and Prism 5.0 software (GraphPad). Rank-sum tests were used to compare the rates between the two groups of immunohistochemistry data, multisample average one-way ANOVA tests were used for knockdown group comparisons, and Student’s t-tests were used for overexpression experiment data. α = 0.05 indicated statistical significance. *P* < 0.05 was considered statistically significant. n.s. was indicated in the figures when *P* > 0.05, * when P < 0.05, **when *P* < 0.01 and *** when *P* < 0.001.

## Results

### Elevated expression of matrix metalloproteinase 7 in tongue squamous cell carcinoma is correlated with a poor patient clinical outcome

**Fig. 1:**
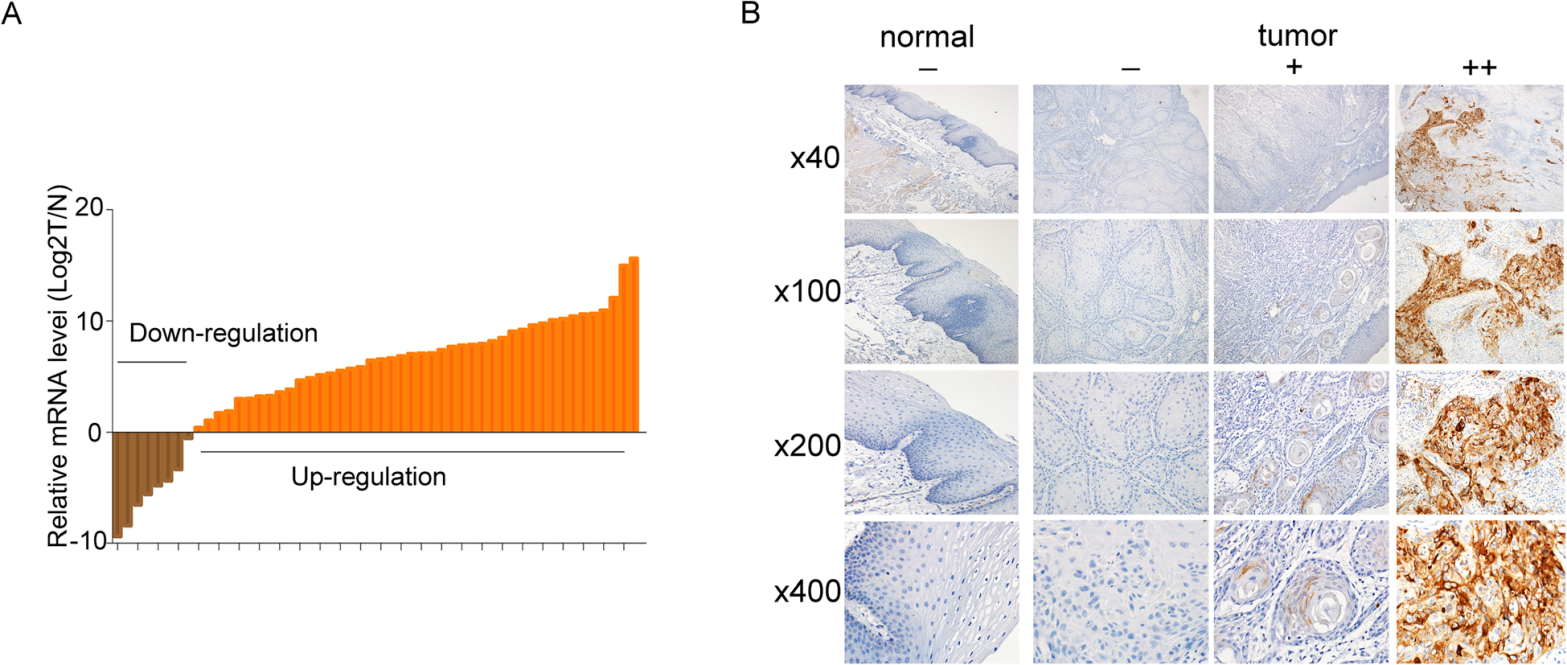
MMP7 is upregulated in tongue squamous cell carcinoma. **a**, The MMP7 mRNA levels in tongue tumours and respective adjacent nontumour tissues were tested by real-time PCR. The ratios of MMP7 in tongue tumour tissues compared with those in the respective nontumour tissues (T/N) from 53 patients are shown. **b**, Representative images of MMP7 protein expression in tongue tumour tissues and adjacent nontumour tissues by immunohistochemistry (−: negative; +: weakly positive; ++: strongly positive)

To investigate the expression pattern of MMP7 in tongue squamous cell carcinoma, real-time PCR was utilized to measure the mRNA expression level. Overexpression of MMP7 was detected in 45 of 53 (84.9%) TSCC samples (Fig. 1a) compared with their respective adjacent nontumour tissues. At the protein level, 88 TSCC specimens containing paired adjacent nontumour specimens, were collected to quantify MMP7 expression by immunohistochemical staining (Fig. 1b). 46 (52.3%) paraffin-embedded cancer specimens were scored as having positive MMP7 expression, and 42 (47.7%) samples showed no MMP7 expression. By sharp contrast, all the paired adjacent normal samples showed no detectable MMP7 protein expression (Table 3). To explore the relationship between MMP7 protein expression and patient clinical characteristics, we analyzed the connection between the expression quantity and patient clinical features, and found that elevated MMP7 expression was associated with lymph node metastasis (*P* = 0.0418, Table 3), but had not significant correlation with tumour stage, differentiation. These data demonstrated that overexpression of matrix metalloproteinase 7 is associated with tumourigenesis and lymph node metastasis of tongue cancer.

**Table 3:** The expression of MMP7 in tongue cancer samples with different clinical and pathological characters

Characteristics	Cases	MMP7+	MMP7-	*P* value
Cancer vs Normal				
Cancer	88	46	42	< 0.001***
Normal	88	0	88	
Gender				
Female	43	21	22	0.6697
Male	45	25	20	
Age				
Less than 55	49	23	26	0.2894
55 and up	39	23	16	
Tumor Stages^a^				
T1 and T2	26	17	9	1.000
T3 and T4	16	10	6	
Differentiation				
Poorly and Moderately	30	17	13	0.6541
Well	58	29	29	
Lymph Node metastasis				
N0	74	35	39	0.0418*
N1 and N2	14	11	3	

^a^ Some samples were lack of the data of tumor stages

* *P* < 0.05; *** *P* < 0.001

Thus, MMP7 expression was exceedingly higher in tongue squamous cell carcinoma both at the mRNA and protein levels than in the respective nontumour tissues, suggesting that MMP7 might play an oncogenic role and a guide to warrant further investigation.

### Effect of MM7 on tongue cancer cell proliferation in vitro

**Fig. 2:**
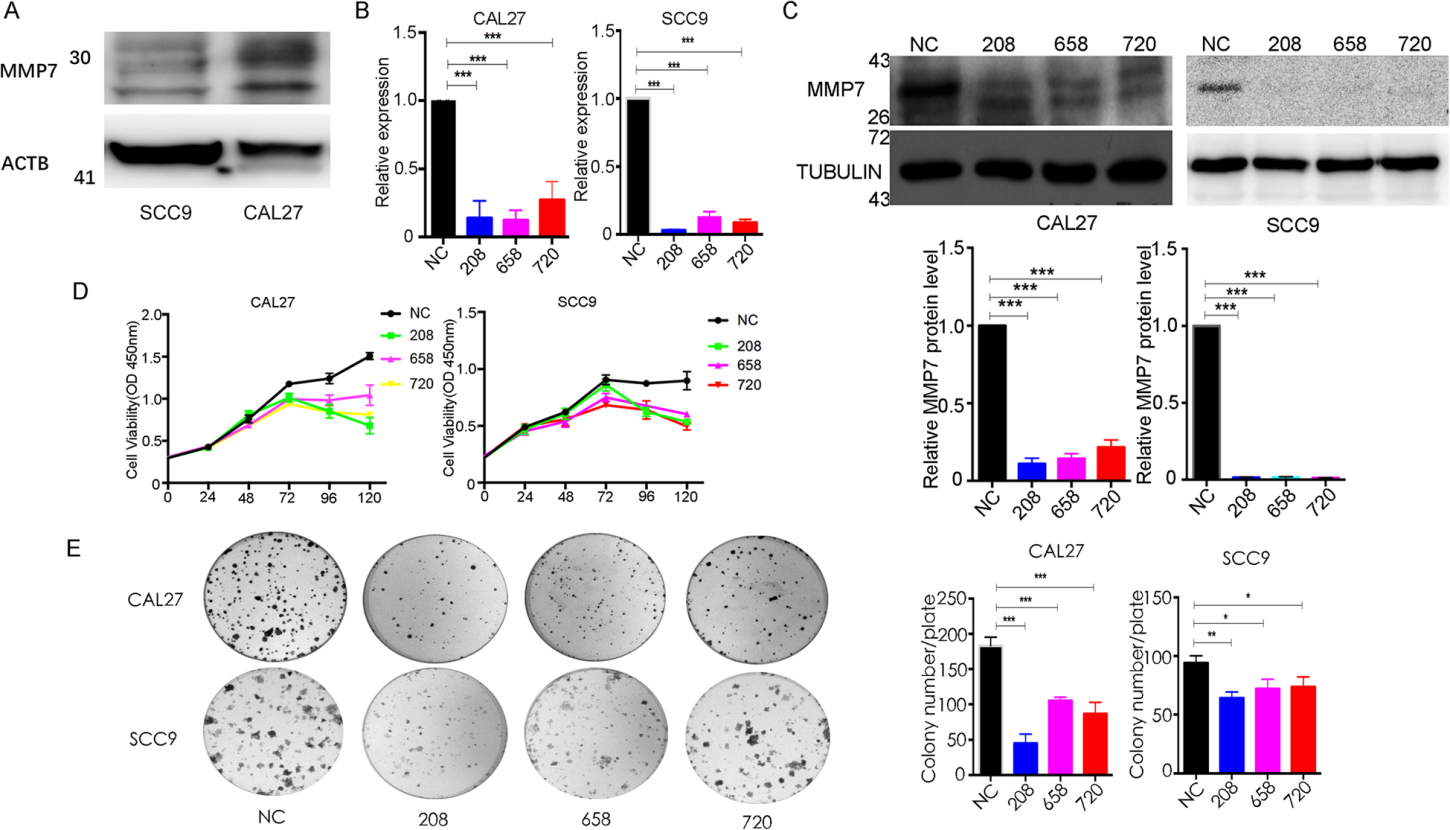
Knockdown of MMP7 inhibits tongue cancer cell proliferation in vitro. **a**, The expression of MMP7 in CAL27 and SCC9 cells were detected by Western blotting. **b**-**c**, The MMP7 expression changes were confirmed by real-time PCR (**b)** and Western blotting (**c)** in the tongue cancer cells (CAL27 and SCC9) after transfecting siRNAs. **d**-**e**, The proliferation ability of tongue cancer cells was measured by the CCK8 assay (**d**, *p* < 0.01 from 72 h to 120 h) and colony formation assay (**e)** after knocking down MMP7. These experiments were repeated three times independently. * when *p* < 0.05, ** when *p* < 0.01, *** when *p* < 0.01

Because MMP7 was upregulated in TSCC and had clinical relevance, we explored whether MMP7 could accelerate the malignant behavior of tongue cancer cells in vitro. First, we measured the expression of endogenous MMP7 in two tongue cancer cell lines: SCC9 and CAL27 and found it to be relatively highly expressed in CAL27 while lower in SCC9 cells (Fig. 2a). To specifically knock down or overexpress MMP7, the corresponding siRNA or plasmid (pCDH-CMV-MCS-EF1–Puro-MMP7) was transfected into the TSCC cell lines CAL27 and SCC9. First, regarding the silencing strategies, the results of real-time PCR (Fig. 2b) and Western blotting (Fig. 2c) demonstrated that MMP7 was knocked down successfully, owing to the lower expression levels of MMP7 in the siRNA-208, siRNA-658 and siRNA-720 groups than those in the negative control group. As shown in Fig. 2d-e, the proliferative abilities of CAL27 and SCC9 cell lines were significantly inhibited after MMP7 was silenced, as demonstrated by CCK8 (Fig. 2d, about 40–50% inhibition, *P* < 0.01 at 96 h and 120 h for both cell lines) and colony formation assays (Fig. 2e, *P* < 0.001 for CAL27 and *P* < 0.05 for SCC9 cells). In the colony formation assay, the effect of MMP7 knockdown in SCC9 (only 30% inhibition) was lower than that in CAL27 cells (> 50% inhibition) which may be due to the lower expression level of endogenous MMP7 (Fig. 2a).

**Fig. 3:**
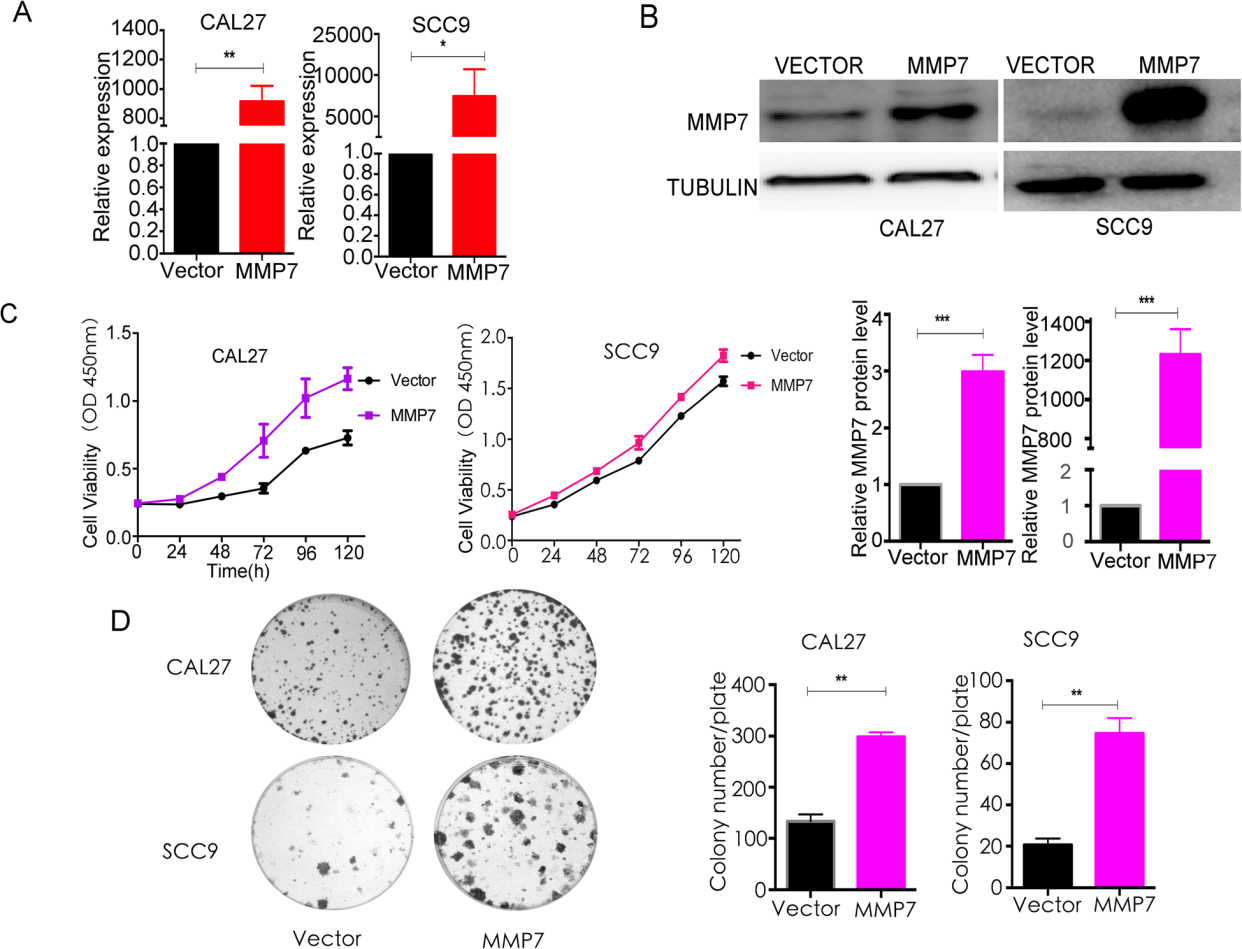
Overexpression of MMP7 promotes tongue cancer cell line growth in vitro. **a**-**b**, After transfection of the overexpression plasmid in CAL27 and SCC9 cells, the expression changes in MMP7 were tested by real-time PCR (**a)** and Western blotting (**b)**. **c**-**d**, The proliferation ability of tongue cancer cells was measured by the CCK8 assay (**c**, *p* < 0.05 from 48 h to 120 h) and colony formation assay (**d)** after MMP7 overexpression. These experiments were repeated three times independently. * when *p* < 0.05, ** when *p* < 0.01, *** when *p* < 0.01

Additionally, enhanced MMP7 expression significantly promoted the cell growth of CAL27 and SCC9 cells. The results of real-time PCR (Fig. 3a) and Western blotting (Fig. 3b) showed that MMP7 was efficiently overexpressed in CAL27 and SCC9 cells after transfection of the plasmid (pCDH-CMV-MCS-EF1-Puro-MMP7). Overexpression of MMP7 accelerated the proliferative progression of CAL27 and SCC9 cells (Fig. 3c-d), according to the results of CCK8 assay (Fig. 3c, *P* < 0.01 at 48, 72, 96, 120 h for CAL27 cells, and *P* < 0.05 at 72, 96, 120 h for SCC9 cells) and colony formation assay (Fig. 3d, P < 0.01 for both cell lines).

Taken together, the evidence above suggests that MMP7 promotes the proliferation of TSCC cells in vitro.

### MMP7 promotes tongue cancer cell migration and invasion in vitro

**Fig. 4:**
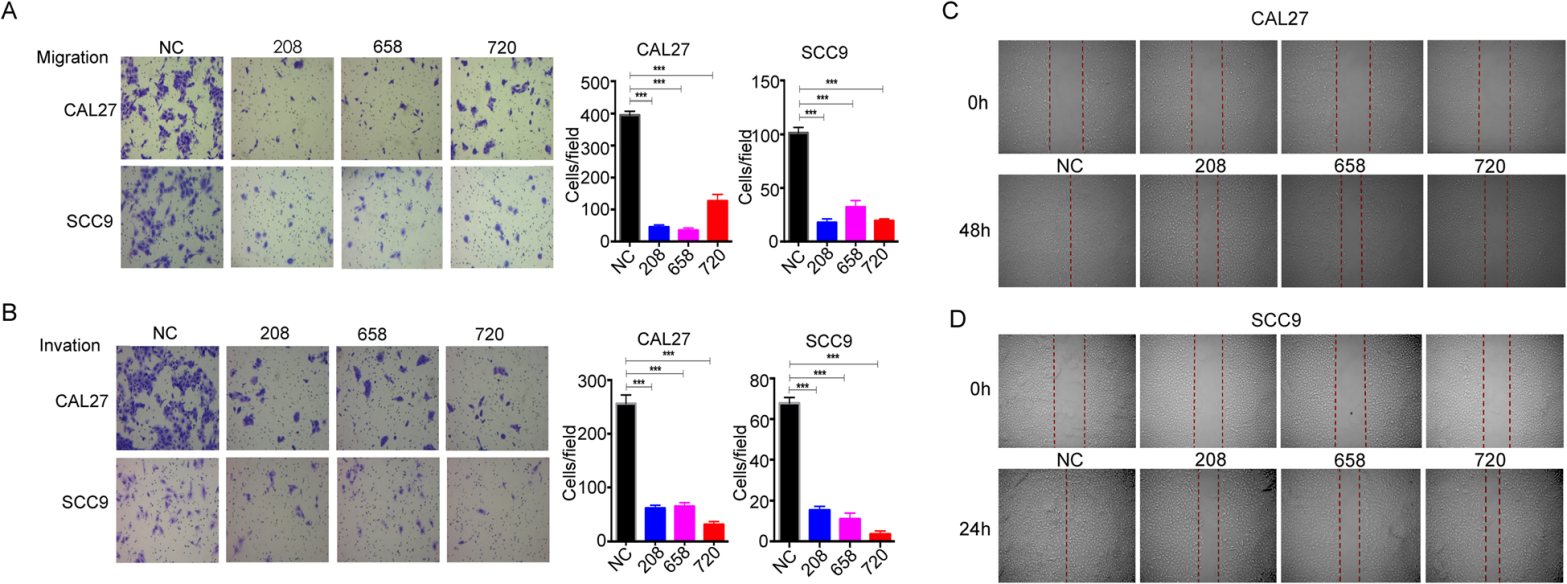
Inhibition of the expression of MMP7 suppresses the tongue cancer cell metastatic potential in vitro. **a**-**b**, Representative images of Transwell chambers coated without (upper panel) or with (upper panel) Matrigel after siRNA transfection, representing the migration and invasion abilities of the CAL27 and SCC9 cells. The number of cells crossing the Transwell chambers were counted. **c**-**d**, Representative photomicrographs (× 40) from the wound healing assay that monitored cell migration are shown. These experiments were repeated three times independently. *** *p* < 0.01

Metastases in TSCC is regarded as one of the most significant factors leading to its relatively poor survival rate. According to previous studies, degrading the components of basement membrane and ECM by MMP7 is a basic step of malignant carcinoma cell migration. Hence, to explore the effect of MMP7 on CAL7 and SCC9 cell migratory behavior, the Transwell assay (in which the carcinoma cells in the chamber migrate across the membrane to the opposite side because of the lack of FBS) was performed. We found that regardless of whether Matrigel was added to the membrane, the number of cells in the siRNA-mediated knockdown groups was much lower and had a statistical discrepancy compared with the negative control group (Fig. 4 A-B, *P* < 0.001 for both cell lines in migration and invasion assays) which showed about 80% inhibition of migration and 70% inhibition of invasion in both cell lines. Additionally, in the wound-healing assay that monitored cell migration for 24 or 48 h, the tracks from the MMP7-silenced group remained stationary or migrated a short distance whereas the negative control cells were almost healed, both in the CAL27 and SCC9 cell lines, displaying a lack of MMP7-restrained motility (Fig. 4c-d).

**Fig. 5:**
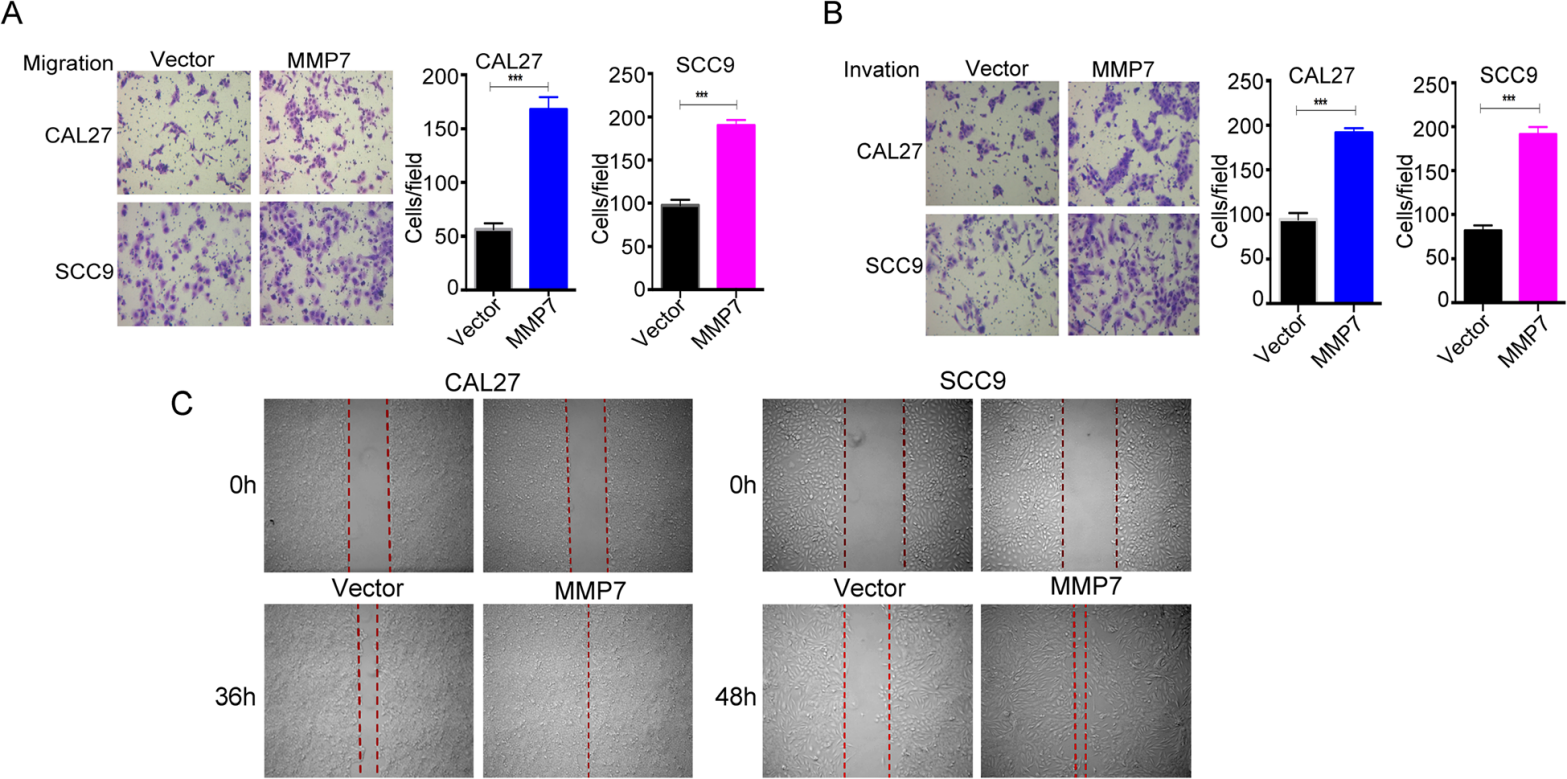
Overexpression of MMP7 promotes the tongue cancer cell metastatic potential in vitro. The cell migration and invasion ability were measured by the Transwell assay with or without Matrigel coating (**a**-**b**) and wound healing assay (**c**). **a**-**b**, Representative images of the cells that crossed the Transwell chambers containing the control and overexpression groups are presented. Quantification of the results is presented as the mean ± SD (***, *P* < 0.001). Wound healing assays were performed, and representative images are shown in **c** (× 40). These experiments were repeated three times independently

However, as shown in Fig. 5c, elevated MMP7 remarkably overlapped the track compared with that in the control groups. In sharp contrast, MMP7 overexpression showed inverse results compared with the knockdown experiment in the migration assay (Fig. 5a, *P* < 0.001 for both cell lines, about 200% increase in CAL27 and 100% increase in SCC9 cells), invasion assay (Fig. 5b, P < 0.001 for both cell lines, 100% increase in CAL27 and SCC9 cells) and in the wound healing assay (Fig. 5c).

All the functional studies intensively indicated that MMP7 could promote the tongue cancer cell motility in vitro and confirmed its oncogenic role in the tumourigenesis and progression of tongue cancer.

### MMP7 knockdown inhibits tongue cancer cell metastasis in vivo

**Fig. 6:**
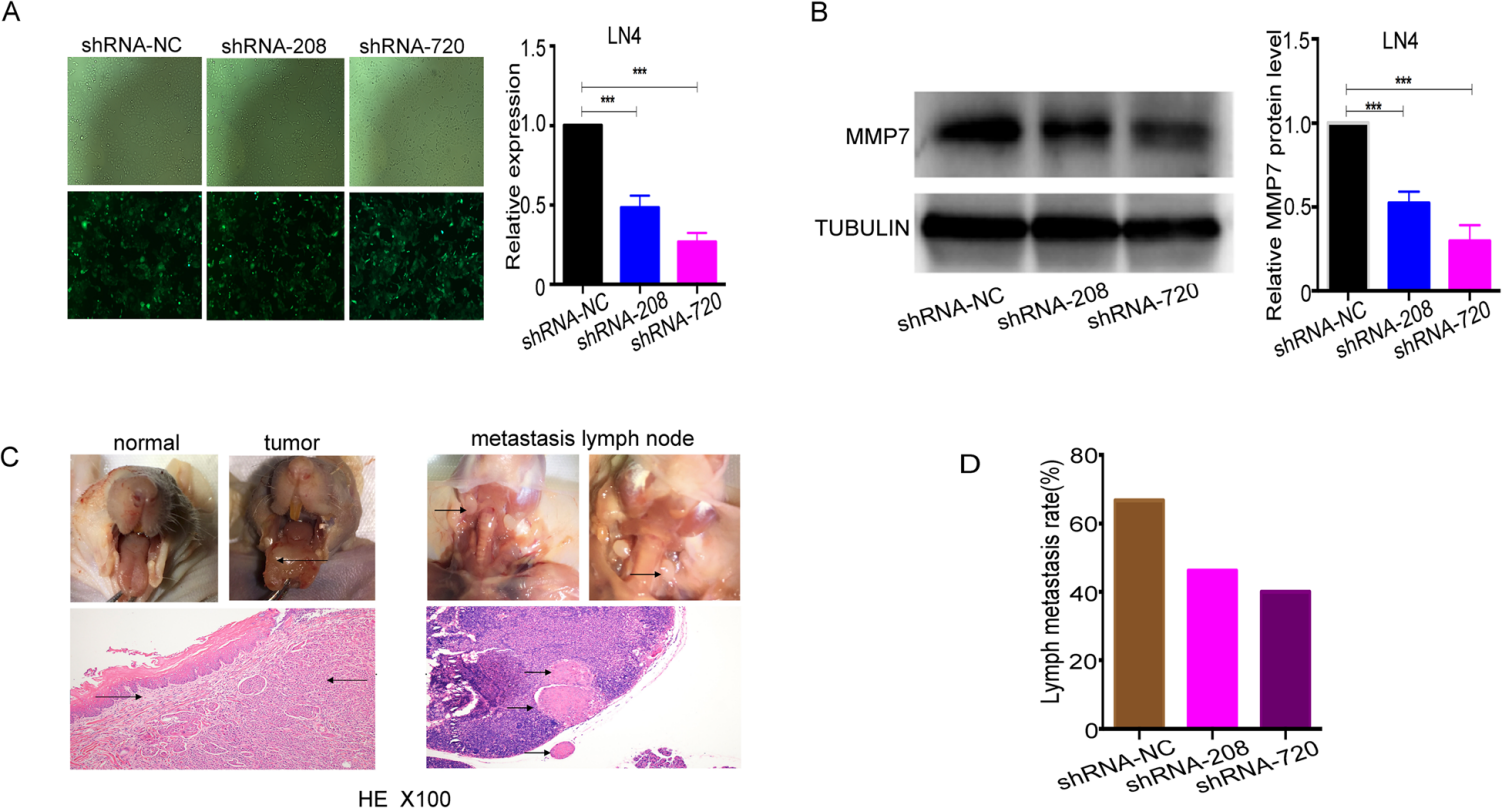
MMP7 knockdown inhibits tongue cancer cell metastasis in vivo. **a**, Representative images of LN4 cells infected with lentiviral vectors or not are shown in **a** (left). The MMP7 mRNA expression levels of the negative control and MMP7-silenced groups were tested by quantitative real-time PCR (**a**, right). **b**, The effect of knocking down was confirmed by Western blotting analysis for these stabilized cell lines. Representative images of the tongue of nude mouse without or with tumour and metastasis lymph node from the neck, confirmed by HE simultaneously, are represented in **c**. The arrows in **c** (right) indicate metastatic lymph nodes. **d**, The statistical result of the lymph metastasis rate from each group is represented

As reported above, we found that MMP7 could accelerate tongue cancer cell migration and invasion in vitro. To explore the effects of MMP7 in vivo, we constructed an orthotopic nude mouse tongue cancer model with silenced MMP7 in a CAL27-derived high-metastasis tongue cancer cell line LN4 (Fig. 6a-b). We observed metastasis in the lymph nodes from the neck in 10 of 15 (66.7%) mice in the negative control group, 6 of 13 (46.2%) mice in the shRNA-208 group, and 6 of 15 (40.0%) mice in the shRNA-720 group (Fig. 6c-d). No cancer cells were detected in the lymph nodes from the oxter, abdomen and popliteal space in all the nude mice. These findings demonstrate that the metastatic potential of tongue cancer cells was reduced in vivo when MMP7 is knocked down, consistent with the conclusions from the molecular function trials in vitro.

## Discussion

Despite the poor prognosis of tongue carcinoma due to its invasion, drug resistance and recurrence and the severe adverse effects on patients’ appearance, swallowing, and pronunciation, surgical resection combined with chemotherapy and radiotherapy have been considered the most effective means to treat this disease for decades. Thus, it is extremely urgent for researchers to explore targeting key molecular biomarker treatment as alternatives. Among the progression steps of malignant squamous cell carcinoma, metastasis is a critical event in which neoplastic cells traverse the basement membranes and extracellular matrix after casting off from the primary nasopharyngeal carcinoma toward the endothelial cells of the vasculature and lymphatics. Many studies have found that the proteolysis of matrix metalloproteinases (MMPs) accelerates this movement by degrading almost all the compounds of the ECM and basement membranes [18].

Among the 24 members of the MMP family, previous researches have focused on the tumourigenic role of MMP9 and MMP2 for many malignant carcinomas [19]. Few papers have reported on MMP7 as a tumour regulator in TSCC, although its function in facilitating tumourigenesis is canonical in many cancers [20–22]. In our study to explore the role of MMP7 in the carcinogenesis and metastasis of TSCC, we found that upregulated MMP7 expression was frequently detected in clinical TSCC samples which was tightly related to increased lymph node metastasis. This discovery was consistent with the report from Barros et al. [23] that high-grade tongue cancer showed elevated expression of MMP7. Further functional assays of TSCC cells in vitro demonstrated that the enhanced expression of MMP7 in the CAL27 and SCC9 cell lines promoted cell proliferation, migration and invasion. However, silencing MMP7 showed the opposite phenomenon. Subsequently, to investigate whether MMP7 functions similarly in vivo, an orthotopic nude mouse model of tongue cancer in which MMP7 is silenced stably in LN4 was constructed. Similarly, a lower metastasis rate was detected in the silenced group, indicating that MMP7 could accelerate the metastasis of tongue squamous cells. However, all the metastatic lymph nodes were from the neck, not the oxter, abdomen and popliteal space. Additionally, the cancer cells were not found in the hearts, livers, and lungs of the nude mice. Thus, this model could not simulate the distant metastasis of tongue cancer and is considered a limitation of this experiment. In our future study, some improvement measures would be taken to solve this problem. Nevertheless, these results clearly illustrated that MMP7 functions as a oncogene in tongue cancer and could be exploited as a meaningful therapeutic target of TSCC.

Additionally, besides proteolysis against ECM and basement membranes, how MMP7 plays its oncogene role in tongue cancer remains elusive. In one proposed mechanism, the imbalance of MMPs and TIMPs, the endogenous inhibitors of MMPs, is believed to be a critical factor for tumourigenesis and metastasis [24, 25]. TIMPs, including the 4 founding members TIMP1/2/3/4, have been verified to function as antagonists against the proteolytic effect from MMPs by stabilizing the basal membrane [26] and restricting the invasion and metastasis of cancer cells. One or more TIMPs can inhibit one type of MMP [27]. J SAFRANEK et al. [28] reported higher expression of MMP7 mRNA and lower expression of TIMP1 mRNA in NSCLC tissue as compared to nontumour lung tissue, offering the first step for further application. Mustafa Gunes et al. [29] reported that the preoperative serum levels of MMP7 were significantly higher in patients with bladder cancer with metastatic disease, lymphovascular involvement, and lymph node metastasis than in control groups, but serum TIMP1 levels showed the opposite result. These investigations indicate that TIMP1 may be an antagonist against the proteolytic effect of MMP7.

Furthermore, many authors have proposed that MMP7 may indirectly destroy the vital components of the extracellular matrix by activating other individual MMPs or associating with other MMPs to promote tumour cell metastasis. Crabbe T et al. [30] and F Q Wang [31] indicated that gelatinases (e.g., MMP2 and MMP9) could be activated by MMP7, and Imai K et al. [32] demonstrated that MMP7 could enhance MMP1 activity and partially activate pro-MMP9 in human rectal carcinoma cells. In agreement with Christoph Wille et al. [33], MMP7 is likely to be the upstream gene of MMP9 in the invasion behavior of pancreatic cancer cells. Additionally, the cooperating expression of MMP2, 7 and 9 may induce colorectal tumour cells to invade locally and distantly or promote new blood vessel formation [34]. However, another investigation showed an increasingly compensatory MMP2 mRNA level in mice in the absence of MMP7 intestinal tumourigenesis [35], indicating a negative correlation between individual MMPs.

The mechanism underlying how MMP7 can promote tumour development in other fields has also earned widespread respect. E-cadherin, a vital intercellular adhesion protein, was confirmed as one of the substrates of MMP7, leading to the detachment and metastasis of malignant cells from the primary lesion [36, 37]. First, CD34, an endothelial progenitor biomarker, was discovered in renal cell carcinoma expressing MMP7 [38], suggesting that MMP7 may contribute to tumourigenesis by correlating with tumour-induced neovascularization. Second, Lionel Remy et al. reported that MMP7 promotes colon carcinoma cell migration via cleavage of the laminin-5 beta3 chain [13]. Additionally, MMP7 was reported as a target gene of the WNT/β-catenin signaling pathway in many carcinomas [39–41], and micro489 [42], state3 [14], and cox-2 [43] were reported as upstream genes of MMP7 in the cooperative function of regulatory carcinomas. Regrettably, the mechanism underlying how MMP7 promotes the proliferation, metastasis and invasion of tongue squamous cell carcinoma was not revealed, although we demonstrated that MMP7 acts as oncogene in TSCC, a finding that will push us to research further.

Because so many studies have confirmed the carcinogenesis of MMP7, the diagnosis, prognosis, treatment and prevention of malignant tumour patients targeting MMP7 were explored to improve the unfavorably poor clinical outcome. Tao Jiang et al. [44] showed that the number of circulating anti-matrix metalloproteinase 7 antibodies was elevated in patients with oral squamous cell carcinoma compared with that in normal controls and its higher levels were significantly correlated with lower histological differentiation, lymph node metastasis, late TNM stage and poor overall patient survival. Ramankulov et al. [45] confirmed this finding by measuring the plasma MMP7 levels in patients with renal cell carcinoma. Additionally, higher MMP7 expression was found both in the cancer tissues and sera of colorectal patients compared with the control group, leading to distant metastasis of cancer cells. For treatment against MMP7 carcinogenesis, Y J Fang et al. [46] discovered that endocrine therapy was effective in restraining ERβ-positive colon cancer cell proliferation and migration via the downregulation expression of MMP7. It was also reported that MMP7 was associated with the acquisition of the chemosensitivity of 5-FU [47] and doxorubicin [48] because of its role in tumour cell escape from Fas-mediated apoptosis. Furthermore, Gang Zheng et al. [49] offered preliminary evidence for MMP7-triggered photodynamic therapy efficacy in cancer treatment, although synthetic metalloproteinase inhibitors targeting MMPs revealed disappointing results in human clinical trials [50]. Additionally, MMP7 was initially validated as a precancerous potential biomarker to prevent colon carcinoma [51]. Interestingly, Tie-Jun Li et al. [43] reported that the expression of MMP7 in oral cancer tissues was highly elevated compared with that in oral lichen planus (OLP); MMP7 expression in OLP was significantly higher than that in the normal oral mucosa at both the mRNA and protein levels, suggesting that MMP7 might be an early precancerous indicator for OSCC owing to the confirmation of OLP as the canonical precancerous lesion. These papers deeply inspired us to explore further the diagnosis, treatment and prevention role of MMP7 in tongue carcinoma.

## Conclusion

Herein, all the data in our study, despite the limitations, demonstrate that MMP7 plays an oncogenic role in the tumourigenesis and metastasis of tongue cancer by promoting malignant cell proliferation, migration and invasion. Therefore, MMP7 may be regarded as a prospective therapeutic target to cure tongue cancer patients.

## Data Availability

All data generated or analyzed during this study are included in this published article.

## Abbreviations

CCK8: Cell Counting Kit-8
ECM: Extracellular matrix
MMP: Matrix metalloproteinases
NC: Negative control
OLP: oral lichen planus
OSCC: Oral squamous cell carcinoma (OSCC)
TSCC: Tongue squamous cell carcinoma
